# Surface-anchored poly(acryloyl-L(D)-valine) with enhanced chirality-selective effect on cellular uptake of gold nanoparticles

**DOI:** 10.1038/srep31595

**Published:** 2016-08-17

**Authors:** Jun Deng, Sai Wu, Mengyun Yao, Changyou Gao

**Affiliations:** 1 MOE Key Laboratory of Macromolecular Synthesis and Functionalization, Department of Polymer Science and Engineering, Zhejiang University, Hangzhou 310027, China

## Abstract

Chirality is one of the ubiquitous phenomena in biological systems. The left handed (L-) amino acids and right handed (D-) sugars are normally found in proteins, and in RNAs and DNAs, respectively. The effect of chiral surfaces at the nanoscale on cellular uptake has, however, not been explored. This study reveals for the first time the molecular chirality on gold nanoparticles (AuNPs) functions as a direct regulator for cellular uptake. Monolayers of 2-mercaptoacetyl-L(D)-valine (L(D)-MAV) and poly(acryloyl-L(D)-valine (L(D)-PAV) chiral molecules were formed on AuNPs surface, respectively. The internalized amount of PAV-AuNPs was several times larger than that of MAV-AuNPs by A549 and HepG2 cells, regardless of the chirality difference. However, the D-PAV-AuNPs were internalized with significantly larger amount than the L-PAV-AuNPs. This chirality-dependent uptake effect is likely attributed to the preferable interaction between the L-phospholipid-based cell membrane and the D-enantiomers.

The design of smart multifunctional nanoparticles (NPs) for targeted therapies and intracellular imaging requires insight understanding of cellular uptake of NPs and their intracellular fates^1^,^2^. For clinical and biological applications, controlling and manipulating the accumulation of NPs for an extended period of time inside cells can achieve improvements in diagnostic sensitivity and therapeutic efficiency^3^.

NP uptake begins with an initial adhesion of the NP to cell membrane and the interaction with integral proteins, polysaccharides, lipids, and other components of the cell membrane. The cellular uptake is an energy-dependent uptake process^4^,^5^, allowing internalization of NPs^4^,^6^. One of the key steps in NP uptake is therefore the very initial interaction. From a viewpoint of chemistry, the cell membrane is composed of phospholipid bilayers integrated with proteins and polysaccharides^7^. As an amphiphilic molecule containing a hydrophilic head and a hydrophobic tail, the phospholipid possesses the chiral nature, showing the L-enantiomer (Fig. 1). The amino acids in proteins of the membrane, except of glycine, are “left-handed”, whereas all the sugars in polysaccharides of the cell membrane are based on the “right-handed” sugar ring^8^ (Fig. 1). The highly ordered arrangement of these molecules endows the membrane with an apparent asymmetric feature, which is one of the predominant biochemical signatures of life. Many chiral superstructures can be self-assembled from chiral or achiral molecules, and these chiral superstructures may be used in various fields as templates for helical crystallization, molecular recognition, catalysis and so on^9^,^10^,^11^,^12^. Recently, pioneering works have been conducted to reveal the cell behaviors such as cell adhesion^13^ and differentiation^14^, and protein adsorption^15^,^16^ (*e.g.* amount and affinity) on flat substrates anchored with different chiral molecules. Some other works attempted to develop chiral gold nanoclusters (AuNCs) and quantum dots (QDs) with optical activities using different chiral stabilizers for cell imaging^17^. Although the most biological effects of NPs can be linked to their different cellular uptake, little is known on how NP surface chirality at the nanoscale affects the cellular uptake and the successive biological fates. More recently, attend is paid to investigating the cytotoxicity induced by surface chirality at the nano or sub-nano levels^18^,^19^. However, how the NP surface chirality at the nano level influences the cellular uptake has not been explored. These facts inspire us to introduce the surface chirality at the nanoscale and to study the difference in NP uptake from a biomimetic point of view.

Gold nanoparticles (AuNPs) have great potentials as anticancer drug delivery carriers and photothermal cancer treatment agents because of their unique chemical and physical properties (*e.g.* size- and shape-dependent optical and electronic features, high surface-to-volume ratio, excellent biocompatibility and chemical stability)^20^. These properties indicate that AuNPs can act as an ideal platform to investigate the chiral effect on NP uptake, when they are combined with chiral characteristics. In nature most amino acids exist as the L-enatiomers, and the chirality of amino acids strongly influences the steric configurations and higher-order conformations of proteins and other biomacromolecules. Valine is one of the eight essential amino acids of human body, playing essential roles in a wide variety of physiological processes^16^,^21^,^22^,^23^.

In this study the L- and D-valine are selected as the chiral centers, and polymers containing L- and D-valine are prepared to enhance the chiral effect (Fig. 1). For this purpose, small 2-mercaptoacetyl-L(D)-valine (L(D)-MAV) and poly(acryloyl-L(D)-valine) (L(D)-PAV) molecules are synthesized, and are further grafted onto AuNPs to explore the chiral effect on cellular uptake (Fig. 1). Lung and liver are the major organs that NPs will accumulate when they enter into the body. Therefore, lung cells and liver cells are widely used in the *in vitro* cell culture to study cell-NP interactions. This chirality-associated regulation of cellular uptake highlights the important role of the conformation of the stabilizers, and has important medical implications for the design of novel AuNPs.

## Results and Discussion

### Characterization of chiral poly(acryloyl-L(D)-valine) and 2-mercaptoacetyl-L(D)-valine

**Figure 2:**
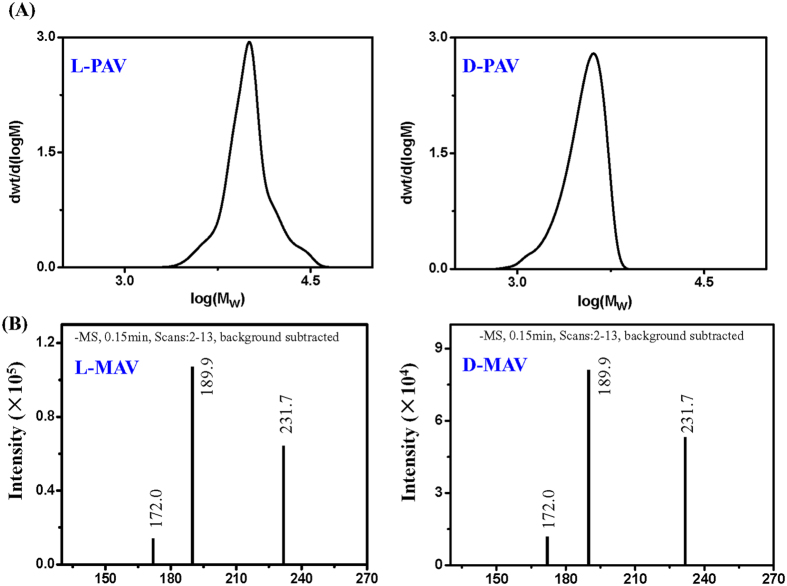
(**A**) GPC curves of (Left) L-PAV and (Right) D-PAV. (**B**) MS spectra of (Left) L-MAV and (Right) D-MAV.

To synthesize the PAV molecules, the monomers of L(D)-acryloylated amino acids were synthesized and polymerized via the reversible addition-fragmentation chain transfer (RAFT) polymerization method. According to GPC, the L-PAV and D-PAV had a similar weight average molecular weight (*M*_w_) of 3580 Da and 3610 Da, and narrow polydispersity (*D*) of 1.14 and 1.15, respectively (Fig. 2A). The small 2-mercaptoacetyl-L-valine (L-MAV) and 2-mercaptoacetyl-D-valine (D-MAV) were successfully synthesized as well: [M-H]^−^: 189.9 for L(D)-MAV and [M-H]^−^: 231.7 for un-reacted 2-(thioacetyl)acetyl-L(D)-valine (Fig. 2B).

### Characterization of AuNPs

**Figure 3:**
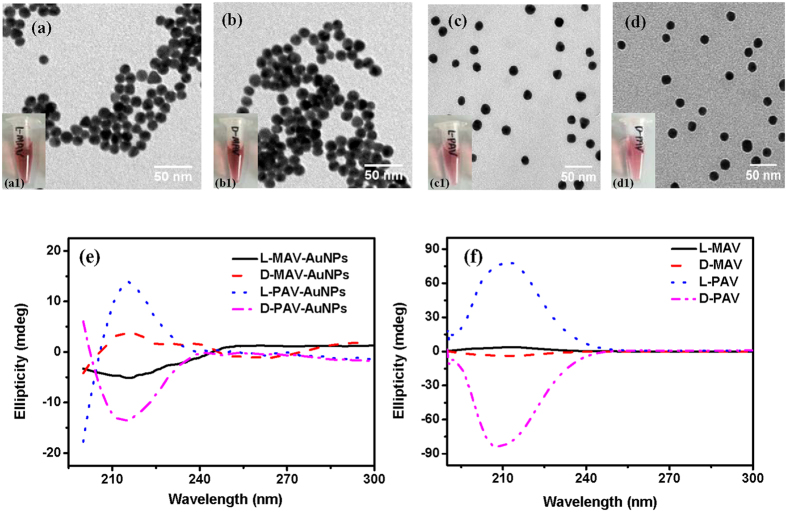
Nanoparticle characterization. TEM images of (**a**) L-MAV-AuNPs, (**b**) D-MAV-AuNPs, (**c**) L-PAV-AuNPs and (**d**) D-PAV-AuNPs. Insets are corresponding digital images. CD spectra of (**e**) L(D)-MAV-AuNPs and L(D)-PAV-AuNPs, and (f) L(D)-MAV and L(D)-PAV molecules. The concentrations of the AuNPs in (**e**) and the MAV or PAV molecules in (**f**) were 10 nM and 15 *μ*M, respectively.

The synthesized small and polymer molecules were grafted onto water-dispersible AuNPs by a simple co-incubation, respectively. Transmission electron microscopy (TEM) showed that both the MAV-AuNPs (Fig. 3a,b) and PAV-AuNPs (Fig. 3c,d) were approximately spherical in shape and narrowly distributed (Figure S1).

The density of MAV and PAV molecules on the AuNPs being calculated from TGA (Figure S2) was 2.4 molecules/nm^2^ and 0.8 molecules/nm^2^, respectively. However, the density of AV unit on the PAV-AuNPs (16.2 units/nm^2^) was significantly larger than that on the MAV-AuNPs (2.4 units/nm^2^). The CD signal of PAV-AuNPs was significantly stronger than that of MAV-AuNPs because of more AV units on the PAV-AuNPs (Fig. 3e). Importantly, the L-PAV-AuNPs and D-PAV-AuNPs (or the L-MAV-AuNPs and D-MAV-AuNPs) showed essential mirror image CD spectra in the region of 190 to 300 nm (Fig. 3e). Although the free L- and D-MAV molecules showed mirror CD spectra, their CD direction was reversed after immobilization onto the AuNPs (Fig. 3f). Such reversal of CD signals after small molecule adsorption onto NP surface has been reported previously^24^,^25^.

The more negative surface zeta potential of PAV-AuNPs in water (−36 mV) than that of the MAV-AuNPs (~ −26 mV) is consistent with the larger number of carboxyl groups (more AV units) on the PAV-AuNPs (Table 1). Moreover, the hydration diameter of PAV-AuNPs (~25 nm) was significantly smaller than that of MAV-AuNPs (~35 nm), revealing that the MAV-AuNPs were aggregated to a larger degree in water. Furthermore, the AuNPs stability was monitored by measuring the AuNPs surface plasmon resonance (SPR), a parameter that is extremely sensitive to nanoparticle size and interparticle spacing^26^. A slight red-shift (4 nm) was observed in the SPR peaks of PAV-AuNPs compared to that of the citrate-AuNPs, without significant peak broadening (Figure S3a). By contrast, the MAV-AuNPs underwent a red shift of 13 nm, accompanying by slight peak broadening, which are indicative of slight aggregation (Figure S3a). Compared with the small molecules, the polymer chains with a steric bulk can cap the gold cores more effectively to enhance the colloidal stability of NPs, leading to better dispersion in medium^14^.

The stability of the NPs in 10% FBS/DMEM, in which cells were cultured, was further characterized. The SPR peak of PAV-AuNPs was kept at 521 nm without change, whereas the SPR peak of MAV-AuNPs was slightly blue-shifted (9 nm) (Figure S3b,c). The diameter change was further examined by TEM. The MAV-AuNPs were better dispersed after adsorption of proteins by incubation in FBS containing medium (Figure S4a,b,e,f), whereas the dispersion of PAV-AuNPs was not affected by the medium significantly. These results are well consistent with the SPR results (Figure S3b,c). Moreover, the diameters of the MAV-AuNPs and PAV-AuNPs before and after protein adsorption showed no significant difference in a dry state, which is consistent with previous observation^27^. Hence, the hydrodynamic diameters of the MAV-AuNPs and PAV-AuNPs being incubated in 10% FBS/DMEM were measured by DLS (Table 1). In 10% FBS/DMEM, the hydrodynamic diameters of MAV-AuNPs and PAV-AuNPs were both 38 nm without significant difference, and their surface charge became less negative (Table 1) due to the adsorption of serum proteins including albumin, fibrinogen, and immunoglobulin etc. Therefore, both MAV-AuNPs and PAV-AuNPs possess excellent colloidal stability in 10% FBS/DMEM, and their surface charge and hydrodynamic diameters had no significant difference.

With these characterizations, one can conclude that the L-PAV-AuNPs and D-PAV-AuNPs, or the L-MAV-AuNPs and D-MAV-AuNPs have identical physicochemical properties except of the reverse molecular chirality, respectively. The PAV-capped AuNPs show significantly enhanced chirality that those MAV-capped ones.

### Cellular uptake

**Figure 4:**
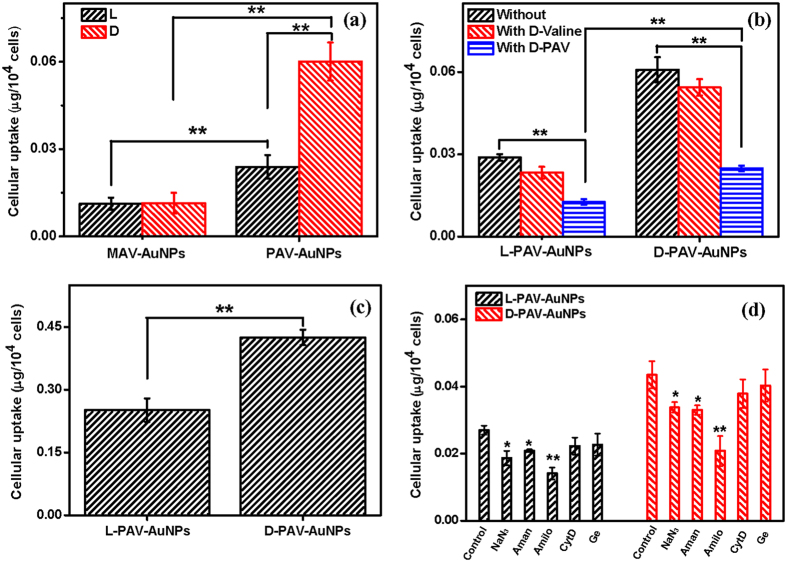
Internalized amount by A549 cells at an Au concentration of 50 *μ*g/mL. (**a**) L(D)-MAV-AuNPs and L(D)-PAV-AuNPs in 10% FBS/DMEM, (**b**) PAV-AuNPs pretreated with 1 mg/mL D-valine or D-PAV (*M*_w_: 18743 Da) in 10% FBS/DMEM, and (**c**) PAV-AuNPs in serum-free DMEM after co-incubation for 24 h. (**d**) Influence of pharmacological inhibitors on uptake of L-PAV-AuNPs and D-PAV-AuNPs, respectively. The cells were cultured without or with pretreatment by amantadine-HCl (Aman, 1 mM, inhibitor of clathrin-mediated endocytosis), genistein (Ge, 100 μM, inhibitor of caveolaemediated endocytosis), amiloride-HCl (Amilo, 2 mM, inhibitor of macropinocytosis), cytochalasin D (CytD, 10 μg·mL^−1^, inhibitor of cytoskeleton), NaN_3_ (0.1% (*w*/*v*), inhibit energy-dependent process) for 1 h, and then cultured with PAV-AuNPs for another 4 h. * and ** indicate significant difference at *p* < 0.05 and *p* < 0.01, respectively.

Next, the cellular uptake of these chiral molecules-capped NPs was investigated by using A549 cells and HepG2 cells. First, MTT assay was performed to evaluate the cytoviability, which reflects the cell metabolic activity based on the ability of mitochondrial succinate/tetrazolium reductase system in living cells. According to literatures and our experiences, the highest cellular loading of NPs such as thiol acids-protected AuNPs and poly(lactic-co-glycolic acid) (PLGA NPs) could be achieved within 24 h^28^,^29^, and some cationic NPs are rapidly internalized within a few hours^30^,^31^. After the cells were treated with PAV-AuNPs at an Au concentration of 40 *μ*g/mL, no significant cytotoxicity was found for the A549 and the HepG2 cells (Figure S5a,b). When the Au concentration of PAV-AuNPs increased to 80 μg/mL, the cytoviability was still above 80% though cell toxicity was significant for the A549 cells (*p* < 0.01) (Figure S5a). No cytotoxicity was observed for the HepG2 cells (Figure S5b). These results suggest that the AuNPs represent low toxicity to cells. Therefore, 50 *μ*g/mL of AuNPs (MAV-AuNPs or PAV-AuNPs) was used to study the cellular uptake, which showed no significant cell toxicity for both A549 and HepG2 cells. The cellular uptake amount of AuNPs (including minor amount of cell-surface attached) was quantified by ICP-MS with high detection sensitivity. Figure 4a shows that the internalized amount of PAV-AuNPs was significantly larger than that of MAV-AuNPs (*p* < 0.01) regardless of the chirality, whereas the amount of internalized L-MAV-AuNPs and D-MAV-AuNPs by A549 cells had no significant difference (*p* > 0.05). The non-influence of small chiral molecules reveals the very weak chirality effects, which is consistent with previous finding that the uptake of L- and D-peptides has no significant difference^32^,^33^. By sharp contrast, the AuNPs capped with PAV of a larger number of AV repeating unit per area did show the chirality-dependent uptake by A549 cells: the internalized amount of D-PAV-AuNPs was nearly 2.6 times higher than that of the L-PAV-AuNPs (Fig. 4a). The internalized amount of MAV-AuNPs and PAV-AuNPs by HepG2 cells had the similar trend to the A549 cells (Figure S6a). Moreover, the relatively larger uptake of D-PAV-AuNPs over L-PAV-AuNPs (*p* < 0.01) was observed at different NPs concentration (10–100 *μ*g·mL^−1^) (Figure S7), although the absolute amount for both types of NPs increased along with the increase of NPs concentration. These results suggest that the chiral effects on cellular uptake can be enhanced by the polymerization of chiral monomers, and the PAV polymers with stronger ellipticity than their smaller counterparts enhance the chirality-dependent interaction with cells, leading to significantly higher uptake of the PAV-AuNPs than MAV-AuNPs.

The chirality-dependent NP uptake is likely tied with the feature of cell membrane. One possibility is that a larger number of targeted receptors exist on the A549 and HepG2 cells (the typical cancer cells) and specifically interact with the D-enantiomers than the L-enantiomers. To testify this hypothesis, A549 cells and HepG2 cells were pre-treated with D-valine for 1 h before incubated with the PAV-AuNPs to block the possible receptors on the cells, which specifically interact with D-enantiomers. Figures 4b and S6b show that the pre-treatment with D-valine impaired the NP cellular uptake to some extent, but the level was insignificant (*p* > 0.05) regardless of the surface chirality. The polymer structure may play a role in the interaction of PAV-AuNPs and cells. Thus, A549 cells and HepG2 cells were pre-treated with D-PAV for 1 h before incubated with the PAV-AuNPs. Figures 4b and S6b show that the internalized amount of AuNPs was largely decreased (*p* < 0.01) regardless of the surface chirality. These results suggest that the larger uptake of the D-enantiomers is likely attributed to the specific receptors or biomolecules in A549 or HepG2 cells that can recognize the right-handed polymers rather than the small counterparts. In fact, the investigation on the interaction between surface chiral molecules (*e.g*. peptides with different chiral amino acid groups) and cells has not found chirality-dependent cell receptors so far^32^,^34^,^35^,^36^. Hence, some other factors or biomolecules in the cells may take the role on the chirality-dependent cellular uptake.

Upon contact with biological fluid, the NPs are rapidly covered by biomolecules, especially proteins, forming a biomolecular corona that effectively screens the bare NP surface^2^. The surface-adsorbed proteins can interact with the receptors of cell membrane, and thereby influence the following cellular uptake. Wang *et al*. have found that surface chirality could influence the protein adsorption^16^. Zhou *et al*. found that serum proteins play an important role in mediating the selective adhesion of cells on chiral surfaces^3^. To clarify this point, the internalization experiments were further conducted in a serum-free medium. As shown in Figures S3c and S4, the L-PAV-AuNPs and D-PAV-AuNPs in serum-free medium showed well dispersion too, ruling out the possible influence of NP aggregation on cellular uptake^37^. Figures 4c and S6c show that the cells still ingested much more D-PAV-AuNPs than L-PAV-AuNPs (*p* < 0.01), and the internalized amount was intensively enhanced than that in the serum-containing medium. The reduction of NPs uptake in serum is likely attributed to the decrease of the surface energy of NPs and the nonspecific interactions between NPs and cell membrane as a result of protein adsorption^38^. Figure S8 shows that L-PAV-AuNPs adsorbed significantly larger amount of serum proteins and albumin than D-PAV-AuNPs. When being incubated under the same conditions, for example, in cell culture medium containing 10% FBS or merely albumin, the largely adsorbed proteins are reported to enhance cellular uptake of NPs^39^,^40^,^41^. However, the results here are on the contrary. Nonetheless, these results substantiate the conclusion that the chirality-dependent cellular uptake of PAV-AuNPs is mainly governed by their surface-chirality property, rather than the surface protein corona.

It is known that the extracellular substances can be transported into cells through several different pathways such as transmembrane diffusion, phagocytosis, and receptor-mediated or nonspecific endocytosis^29^,^42^,^43^. To ascertain the uptake mechanisms of the PAV-AuNPs, some special inhibitors were used to pretreat the cells before co-culture. All the inhibitors presented no significant cytotoxicity at the concentration used (Figure S5c,d). Figures 4d and S6d show that the uptake efficiency of the A549 cells and HepG2 cells was significantly blocked (~31% decrease) by addition of 100 *μ*M NaN_3_, revealing the energy-dependent process. Moreover, cellular uptake of both types of PAV-AuNPs was largely blocked by amantadine-HCl that could prevent budding of clathrin-coated pits (*p* < 0.05, *p* > 0.01) (Fig. 4d), and especially by the amiloride-HCl due to the disturbing of Na^+^/H^+^ channels (*p* < 0.01). Therefore, internalization of the PAV-AuNPs by the A549 cells should be mediated by macropinocytosis and clathrin-mediated endocytosis mechanisms, and macropinocytosis takes the major role for both PAV-AuNPs regardless of their surface chirality. For the HepG2 cells, the NP uptake was only significantly inhibited by amiloride-HCl (*p* < 0.01), regardless of the surface chirality (Figure S6d).

### Interactions of surface chiral molecules and lipids

**Figure 5:**
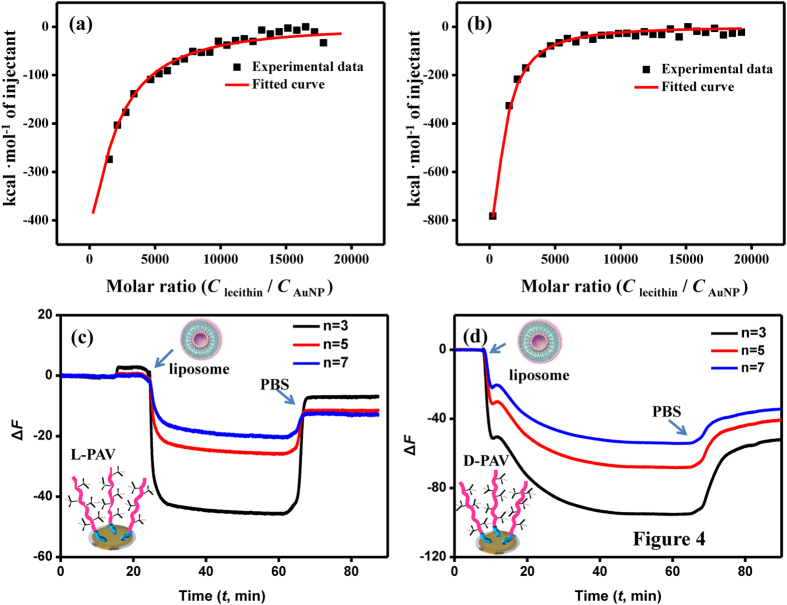
Interactions of surface chiral molecules and lecithin. ITC results showing the evolved heat per mole of added lecithin molecules (corrected for the heat of lecithin dilution) against the molar ratio of (**a**) lecithin/L-PAV-AuNPs and (**b**) lecithin/D-PAV-AuNPs, respectively. The QCM-D results showing the interaction processes between liposomes with (**c**) L-PAV and (**d**) D-PAV. The *n* insets mean the overtone numbers of QCM-D.

The above cellular uptake results suggest that the chirality-dependent endocytosis should be governed by the very initial steps during the uptake, *e.g.* NP adhesion to the cell membrane and interaction with the membrane phospholipids^44^. However, the adhesion of NP to the cell membrane is difficult to disentangle in the presence of simultaneous internalization. Thus, L-lecithin molecules, a type of phospholipids and composed of phosphoric acid with choline, glycerol or other fatty acids, were used as a model to study the interactions of chiral PAV molecules and lipids by isothermal titration calorimetry (ITC)^45^. As shown in Fig. 5a,b, the complexion of L-lecithin with L-PAV-AuNPs and D-PAV-AuNPs was consistently exothermic throughout the titration process. Of all the heat profiles, both the complexion of L-lecithin with L-PAV-AuNP and D-PAV-AuNPs could be satisfactorily fitted by a single set of binding sites available model^46^, and best-fit parameters were calculated by using nonlinear least-squares fitting. The results (Table 2) show that the interaction of L- and D-PAV-AuNPs and L-lecithin featured a favorable enthalpy change (Δ*H* < 0), which was offsetted partially by an unfavorable entropy loss (Δ*S* < 0), resulting in an overall negative free energy change (Δ*G* < 0). Moreover, the complex stability constant *K* value for the D-PAV-AuNPs (9.86 × 10^3^ M^−1^) was about 2.4 folds higher than that for the L-PAV-AuNPs (4.04 × 10^3^ M^−1^), revealing a remarkably stronger affinity for the D-PAV-AuNPs to L-lecithin. The maximum ratios of L-lecithin to L-PAV-AuNPs and D-PAV-AuNPs were about 32.1 and 115, respectively. These results indicate that the L-lecithin molecules prefer to interact with D-PAV-AuNPs.

**Figure 6:**
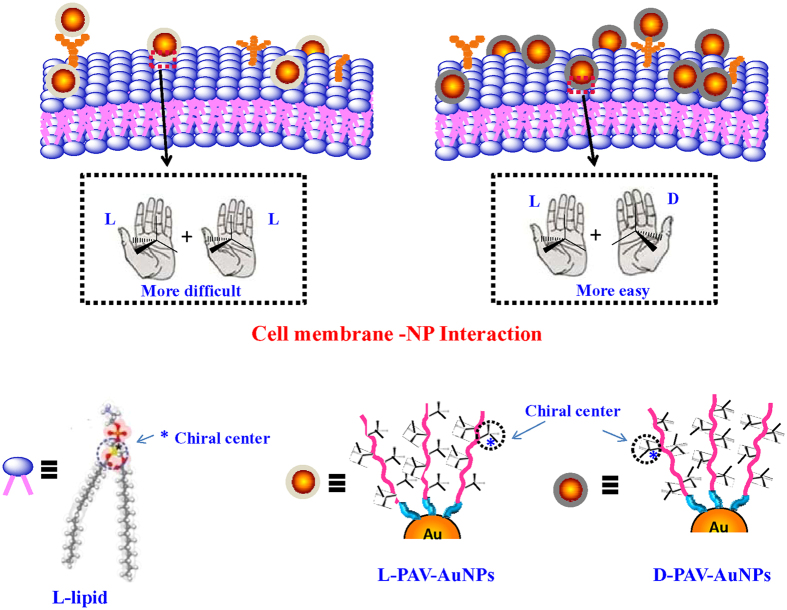
Schematic illustration showing the interactions of NPs and cell membrane. Up, the cell membrane interacts with (Left) L-PAV-AuNPs and (Right) D-PAV-AuNPs, respectively. The double L-lipid bilayers have a stronger interaction with the D-PAV molecules, and thereby the internalized amount of D-PAV-AuNPs is larger. Bottom, the diagram of structure of L-lipid and L(D)-PAV-AuNPs.

In order to further mimic the interaction of surface chiral PAV molecules and cell membrane, a model study was performed by using the surface chiral molecules-immobilized gold-covered electrode and lipid bilayers, whose interaction was monitored by QCM-D. The bilayers composed of L-lecithin were prepared by surface-mediated vesicle fusion^47^. The hydration diameter of vesicles was about 100 nm (number-average). QCM-D can be used to monitor the kinetics of adsorption of small molecules, proteins, or in our case, the interaction with lipid vesicles and chiral PAV molecules grafted on the gold-covered electrode. The lipid vesicles interacted rapidly with the L-PAV (Fig. 5c), whereas rather slowly with the D-PAV (Fig. 5d). As the frequency shift measured by QCM-D is related to the adsorbed mass, more vesicles were attached onto the D-PAV surface than onto the L-PAV surface eventually (Fig. 5c,d). Hence, the L-lecithin-based vesicles prefer to interact with the D-PAV than with the L-PAV. With these results, one can conclude that the left-handed phospholipids-based cell membrane has chiral selective interactions with molecules or NPs. The cell membrane interacts more strongly with the D-PAV-AuNPs than the L-PAV-AuNPs (Fig. 6), leading to larger uptake amount of D-PAV-AuNPs by A549 and HepG2 cells.

**Figure 7:**
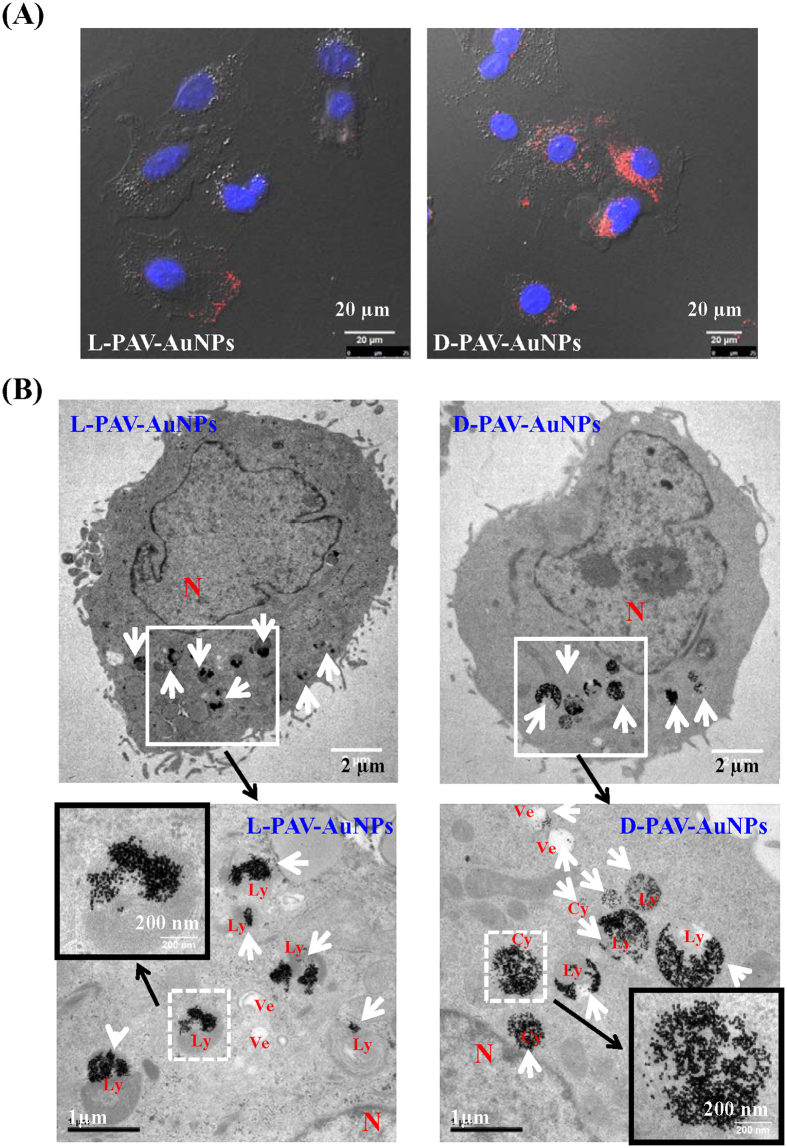
The distribution of NPs in A549 cells. (**A**) CLSM images of A549 cells treated with (Left image) L-PAV-AuNPs and (Right image) D-PAV-AuNPs for 24 h. Merged images of nucleus staining and bright field. (**B**) Representative TEM micrographs of sectioned A549 cells after being cultured with (Left images) L-PAV-AuNPs and (Right images) D-PAV-AuNPs at an Au concentration of 50 *μ*g·mL^−1^, showing subcellular localization of the internalized NPs, respectively. The white arrow heads indicate the PAV-AuNPs, and the black arrows indicate the amplification regions. N: Nucleus; Ly: Lysosome; Ve: Vesicle; Cy: Cytosol.

To further assess the chirality-dependent or independent cellular distribution of PAV-AuNPs, CLSM and TEM (Fig. 7) characterizations were performed. The PAV-AuNPs (red color) were mainly distributed in the cytoplasm, and no signal was detected in the nuclei of A549 cells regardless of the chirality of PAV (Fig. 7). There were more D-PAV-AuNPs (Fig. 7A, Right) than L-PAV-AuNPs (Fig. 7A, Left) in the cytoplasm, and were closer around the cell nuclei (Fig. 7A). TEM observation shows that almost all the L-PAV-AuNPs were located in lysosomes (Fig. 7B, Left). Most of the D-PAV-AuNPs were aggregated in lysosomes too, but some escaped from lysosomes and entered into the cytoplasm as a consequence of larger internalization (Fig. 7B, Right). For HepG2 cells, the L-PAV-AuNPs and D-PAV-AuNPs were mainly distributed in lysosomes regardless of chirality (Figure S7). The cell slice images obtained by TEM here cannot be used to determine the cellular uptake amount of the NPs because the thickness and layers of the cell slice are different.

## Conclusions

This work provides new insights into cellular uptake being triggered by chiral polymers-capped NPs, demonstrating the chirality-dependent uptake amount and intracellular distribution. Two groups of AuNPs modified with MAV and PAV of different chirality were successfully prepared. These two groups of particles had a diameter about 16 nm and narrow size distribution in a dry state. The density of MAV and PAV being grafted on AuNPs was 2.4 molecules/nm^2^ and 0.8 molecules/nm^2^, respectively. Compared to MAV, the PAV grafting endowed the AuNPs with enhanced optical activity due to a larger number of AV units. All the chiral molecules-capped AuNPs possessed good colloidal stability in culture medium, and showed similar physicochemical properties except of reversed ellipticity. While the small chiral molecules-capped L- and D-MAV-AuNPs did not show significant difference in terms of uptake amount by A549 cells and HepG2 cells, the chiral polymers-capped PAV-AuNPs did show chirality-dependent uptake behaviors, and the D-PAV-AuNPs were internalized with significantly larger amount than the L-PAV-AuNPs. The chirality-dependent cellular uptake is likely attributed to the chiral selective interaction between the cell membrane and the chiral PAV on the NPs, as inferred from the fact that the L-phospholipid-based vesicles prefer to interact with the D-PAV molecules. Identification of this chirality-dependent cellular uptake of NPs provides a new idea that chiral effect can act as a novel strategy for designing bio-interface materials and may open a new avenue for further development of AuNPs for biomedical applications.

## Materials and Methods

### Materials

L-lecithin, L-Valine, D-valine, thioacetic, 1-butanethiol, carbon disulfide (CS_2_), ethyl 2-bromopropionate, thioacetic acid and acryloyl chloride were purchased from Aladdin company. Triethylamine (TEA), dimethyl formamide (DMF) and dichloromethane (DCM) were obtained from Sinopharm Chemical Regent Co., Ltd, and were vacuum-distilled prior to use. Gold (III) chloride hydrate (HAuCl_4_), and trisodium citrate dihydrate (C_6_H_5_Na_3_O_7_·2H_2_O) were purchased from Sinopharm group Co. Ld. Amiloride-HCl (Amilo), amantadine-HCl (Aman), sodium azide (NaN_3_), genistein (Ge), Triton X-100, 4′,6-diamidino-2-phenylindole (DAPI), cytochalasin D (CytD) and 3-(4,5-dimethyl-thiazol-2-yl)-2,5-diphenyltetrazolium bromide (MTT) were obtained from Sigma. Acryloyl-L(D)-valine monomers, and methyl 2-(butylthiocarbonothioylthio)propanoate (MCEBTTC) were synthesized and characterized in Supporting information. All other chemicals were of analytical grade and used without further treatment if not specially mentioned. The MilliQ water was used throughout the experiments. For AuNPs synthesis, all glasswares used were cleaned by freshly prepared aqua regia solution (HCl/HNO_3_, 3:1).

### Synthesis and characterization of poly(acryloyl-L(D)-valine)


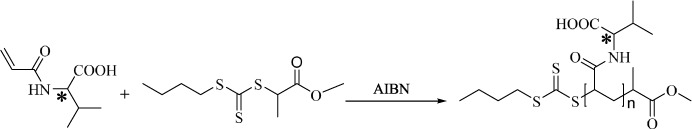


Schematic illustration of synthesis of poly(acryloyl-L(D)-valine) (L(D)-PAV). *Represents chiral center.

Polymerization was carried out in a 10 mL dry Schlenk flask equipped with a magnetic stirrer. Methyl 2-(butylthiocarbonothioylthio)propanoate (50.4 mg), azodiisobutyronitrile (AIBN, 3.4 mg) and acryloyl valine (1.37 g) were dissolved in 5 mL N,N-dimethylformamide (DMF). The mixture was deoxygenated by purging with nitrogen for 20 min, and then heated at 70 °C for 4 h. The reaction was stopped by exposure to air. The mixture was precipitated in excess diethyl ether, and then separated by centrifugation. The dissolution and precipitation cycle was repeated 3 times. The polymers were dried under high vacuum for 48 h at room temperature to give a yellow solid product, which was characterized by gel permeation chromatography (GPC, eluent tetrahydrofuran, Waters 1515 Isocratic HPLC).

### Synthesis and characterization of 2-mercaptoacetyl-L(D)-valine


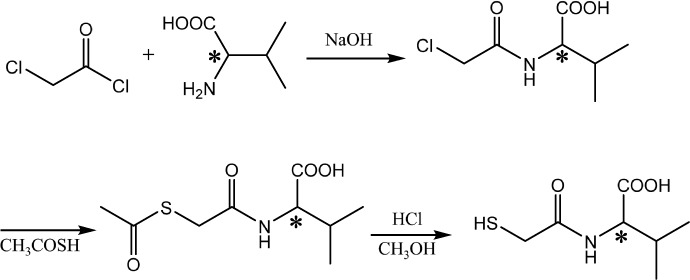


Synthesis of 2-mercaptoacetyl-L(D)-valine (L(D)-MAV).

2-Mercaptoacetyl-L(D)-valine molecules were synthesized using a previously reported method with modification^16^. First, the chloracetyl-L(D)-valine was synthesized by using reagents of chloracetylchloride and L(D)-valine with the similar method mentioned in Supporting Information (Synthesis and characterization of acryloyl-L(D)-valine monomers). 1.1 mL triethylamine and 1.1 mL thioacetic acid were mixed within an ice bath, into which 1 g chloracetyl-L(D)-valine dissolved in 18 mL dichloromethane was added slowly under stirring and nitrogen bubbling. This solution was stirred overnight. After washed 3 times with water, the solvent was removed by vacuum evaporation. The solid product was purified by chromatography on silica gel with ethyl acetate/hexane(*v*/*v* = 30:70) as the eluent. The obtained 2-(thioacetyl)acetyl-L(D)-valine (50 mg) and several drops of HCl solution (1 M) were mixed in 10 mL methanol for 2 h to yield 2-mercaptoacetyl-L(D)-valine, which was characterized by a Bruker Esquire 3000 plus ion trap mass spectrometer (Brucker-Franzen Analytik GmbH, Bremen, Germany).

### Synthesis of AuNPs and surface L(D)-MAV or L(D)-PAV-immobilization

The 15 nm AuNPs were synthesized by a citrate reduction method^48^. The AuNPs solution was centrifuged to remove free citrate molecules, and the concentrated AuNPs solution was collected as a stock solution.

The surface of the obtained AuNPs was functionalized with 2-mercaptoacetyl-L(D)-valine (L(D)-MAV) or poly(acryloyl-L(D)-valine) (L(D)-PAV-AuNPs) using a ligand exchange protocol. Briefly, the stock AuNPs solution was incubated with a large amount of 1 mg/mL L(D)-MAV or L(D)-PAV solution under heavily shaking at room temperature overnight, respectively. The obtained solution was centrifuged (12000 g/min, 60 min) to remove the free MAV or PAV molecules, and followed by dialysis in water for 4 d.

### Characterization of AuNPs

The gold concentration (*m*, g/mL) of the NPs solution and the size of NPs were measured by using inductively coupled plasma mass spectrometry (ICP-MS, Thermo Elemental Corporation of USA, XSeries II) and transmission electron microscopy (TEM, H-7650), respectively^48^.

The circular dichroism (CD) spectrum was recorded by using a JASCO-820 spectropolarimeter equipped with a thermostatically controlled cell holder. The temperature of the sample was controlled at 25 °C. The UV region was scanned between 190 and 400 nm with an average of 3 scans. The concentrations of AuNPs (L(D)-MAV-AuNPs and L(D)-PAV-AuNPs) were kept at 10 nM. The final spectrum (Δ*θ*) of L(D)-MAV-AuNPs and L(D)-PAV-AuNPs was obtained by subtracting the spectrum of same concentration of AuNPs (without MAV or PAV grafting) solution, respectively.

### Dynamic light scattering (DLS) and zeta potential measurement

The Z-average hydrodynamic diameters of the L(D)-MAV-AuNPs and L(D)-PAV-AuNPs were determined at 37 °C in water and 10% fetal bovine serum/Dulbecco’s modified eagle medium (FBS/DMEM) solution by using DLS with a high performance particle analyzer (Zetasizer Nano, Malvern) equipped with a 633 nm wavelength laser, respectively. The scattering intensity was recorded at a 173° angle in kilo counts per second.

The zeta potentials of the L(D)-MAV-AuNPs and L(D)-PAV-AuNPs were measured at pH 7.4 and 37 °C by using the same machine (Zetasizer Nano, Malvern) in water and 10% FBS/DMEM solution, respectively.

### Cellular uptake of AuNPs

The amount of L(D)-MAV-AuNPs and L(D)-PAV-AuNPs internalized by A549 or HepG2 cells was determined by ICP-MS. Briefly, the cells were seeded on a 12-well plate at a density of 5 × 10^4^ cells/cm^2^ and allowed to attach for 24 h. The medium was replaced with 10% FBS/DMEM containing L(D)-MAV-AuNPs or L(D)-PAV-AuNPs with an Au concentration of 50 *μ*g/mL, respectively. After 24 h, the plates were washed 5 times with phosphate buffered saline (PBS) to remove the free AuNPs. After the cells were harvested by trypsinization, their numbers were quantified by a cell counter. The cells were treated with aqua regia (HCl: HNO_3_ = 1:3, volume ratio) for 2 h. The obtained solution was diluted to determine the Au concentration by ICP-MS. The Au amount per 10^4^ cells from ICP-MS analysis is presented as mean ± standard deviation (*n* = 4).

To clarify the uptake mechanism, the energy dependence of cell-NP interaction was assessed by treatment with 0.1% (*w*/*v*) sodium azide (NaN_3_)^49^. Different pharmacological inhibitors, including 2 mM amiloride-HCl (Amilo), 1 mM amantadine-HCl (Aman), 100 *μ*M genistein (Ge), and 10 *μ*g·mL^−1^ cytochalasin D (CytD) were also used to treat the cells for 1 h before incubation with the PAV-AuNPs, respectively. Then the cells were treated with PAV-AuNPs for another 4 h.

To further investigate the chirality-dependent uptake mechanism, the cells were pretreated with 1 mg/mL D-valine and D-PAV for 1 h before incubation with the PAV-AuNPs, respectively. Then the cells were treated with PAV-AuNPs (containing 1 mg/mL D-valine or D-PAV) for another 24 h. The D-PAV used here to pre-treat the cells was polymerized by using the general free radical polymerization (no thioester bond. *M*_w_: 18743 Da; polydispersity: 1.7) to avoid the possible ligand exchange (the thioester bond can bind to the gold surface too).

### Isothermal titration calorimetry (ITC) measurement

The isothermal titration calorimetry measurements were performed by using a thermostated and fully computer-operated isothermal calorimetry (VP-ITC, GE, USA) instrument. All microcalorimetric titrations between L-lecithin and PAV-AuNPs were performed in aqueous solution (water, pH 7.0) at atmospheric pressure and 298.15 K. Each solution was degassed and thermostated by a ThermoVac accessory before the titration experiment. The titration experiment was involved of 30 injections of L-lecithin (titrant, 5 *μ*L per injection from a 10 mM stock L-lecithin solution) at 5 min interval into the sample cell (1439 *μ*L) which contained the 5.6 × 10^−5^ mM PAV-AuNPs (L-PAV-AuNPs or D-PAV-AuNPs) solution. The heat of L-lecithin dilution in the water alone was subtracted from the titration data for each experiment. The data were analyzed to determine the binding stoichiometry (*N*), complex stability constant (*K*), standard molar reaction enthalpy (Δ*H*) and other thermodynamic parameters of the reaction by using the supplied Origin 7.0 software. One set of binding sites model was used to fit the data as reported previously^45^,^50^. The reported thermodynamic parameters were an average of duplicate experiments.

### QCM-D

Liposomes composed of lecithin (Aladdin Company) were prepared from a lipid solution in chloroform (total lipid amount: 15 mg). The solvent was removed under a stream of nitrogen and the resulting lipid film was placed under vacuum at least for 4 h. The dried lipid film was then hydrated in PBS to yield a final concentration of 1000 *μ*M. To form small unilamellar vesicles (SUVs), the lipid solution was sonicated according to the protocols described in literature by using a ultrasonicator (MISONIX Ultrasonic liquid Processors)^44^. The hydration diameter of the SUVs was measured by DLS with a high performance particle analyzer (Zetasizer Nano, Malvern) equipped with a 633 nm wavelength laser. The scattering intensity was recorded at a 173° angle in kilo counts per second.

The interaction with liposomes and chiral PAV molecules was measured by Quartz Crystal Microbalance with dissipation (QCM-D, Q-Sense E4, Sweden) under a flow condition. The PAV molecules were grafted on gold-coated piezoelectric crystals via the strong thiocarbonylthio-Au bond. Briefly, the crystals were incubated in 5 mg/mL PAV molecules (in ethanol) at 37 °C for at least 4 h. Then the crystals were washed with ethanol and water 5 times, respectively.

### Intracellular distribution

For confocal laser scanning microscopy (CLSM, Leica TCS SP5) measurement, the cells were seeded on a glass bottom cell culture dish (diameter, 20 mm) at a density of 5 × 10^3^ cells/cm^2^ and allowed to attach for 24 h. Then, the cells were treated with L(D)-PAV-AuNPs (Au, 50 *μ*g/mL) for another 24 h, before 5 washes with PBS were applied to remove free L(D)-PAV-AuNPs. The cells were fixed with 0.4% paraformaldehyde at 37 °C overnight, and washed with PBS 3 times. The cells were further treated in 0.5% (*v*/*v*) Triton X-100/PBS for 10 min at 37 °C to increase the permeability of cell membrane. After being blocked with 1% BSA/PBS at 37 °C for 2 h, the samples were stained with 4′,6-diamidino-2-phenylindole (DAPI, Sigma, 1:50) and FITC-phallotoxins (Invitrogen, 1:100) at 37 °C for 30 min, following with 5 washes with PBS.

For TEM cell section analysis, the cells were seeded on a 6-well plate at a density of 5 × 10^4^ cells/cm^2^. After cultured for 24 h, the cells were further incubated with the L(D)-PAV-AuNPs (Au concentration 50 *μ*g/mL) for 24 h. The cells were then washed 5 times with PBS, trypsinized, centrifuged, and fixed with 2.5% glutaraldehyde at 4 °C for 2 h. After 3 washes with PBS (10 mM, pH 7.4), the samples were fixed with 1% perosmic oxide for 2 h at 4 °C. After being washed in water, the samples were dehydrated in a series of ethanol solutions with increased concentrations, embedded, and sliced with a thickness of ~50–70 nm.

### Statistical Analysis

The experimental data are expressed as mean ± standard deviation, and the significant difference between groups was analyzed by using one-way analysis of variance (ANOVA) (for two groups) and two-way ANOVA (for more than two groups) in the Origin software. The statistical significance was set as *p* < 0.05 and *p* < 0.01, respectively.

## Additional Information

**How to cite this article**: Deng, J. *et al*. Surface-anchored poly(acryloyl-L(D)-valine) with enhanced chirality-selective effect on cellular uptake of gold nanoparticles. *Sci. Rep.* **6**, 31595; doi: 10.1038/srep31595 (2016).

## Supplementary Material

Supplementary Information

## Figures and Tables

**Figure 1:**
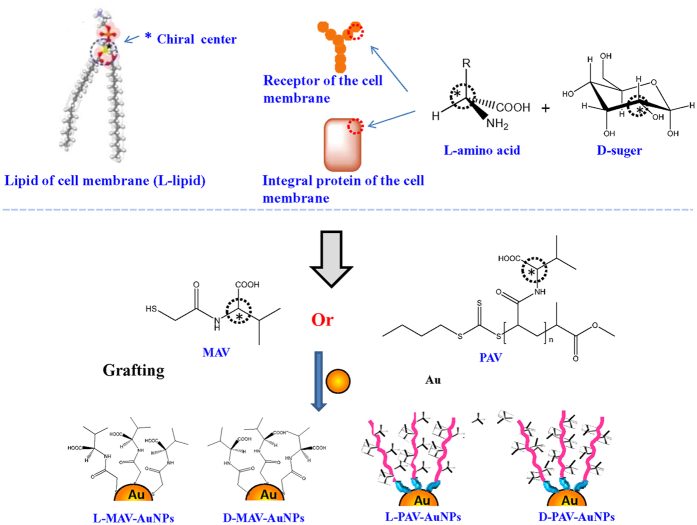
Chiral nature of phospholipid, and amino acid and sugar units in cell membrane inspires the study of influence of chirality on cellular uptake, in which the chiral molecules (MAV and PAV) are grafted onto AuNPs and are used as a platform to study the chirality-dependent cellular uptake.

**Table 1:** Physicochemical properties of AuNPs.

AuNPs	Diameter (TEM, nm)	Molecules density (molecules/nm^2^)	Diameter (DLS, nm)		Zeta potential (mV)	
			Water	10% FBS	water	10% FBS
L-MAV-AuNPs	15.5 ± 1.5	2.47	35.1 ± 3.4	38.1 ± 4.2	−27.4 ± 1.8	−5.8 ± 1.6
D-MAV-AuNPs	15.9 ± 1.8	2.43	34.5 ± 2.1	37.9 ± 1.9	−25.8 ± 0.9	−5.8 ± 1.2
L-PAV-AuNPs	15.9 ± 0.7	0.833#	27.2 ± 1.3	38.2 ± 3.3	−36.4 ± 1.4	−9.1 ± 1.1
D-PAV-AuNPs	16.3 ± 0.6	0.822#	24.1 ± 2.2	34.5 ± 2.3	−36.3 ± 1.6	−9.3 ± 0.8

Note: ^#^Means PAV density. The density of AV units (defined as the repeating chiral unit) on the L-PAV-AuNPs and D-PAV-AuNPs are 16.23 and 16.21 units/nm^2^, respectively.

**Table 2:** Thermodynamic quantities of L-lecithin interaction with L-PAV-AuNPs and D-PAV-AuNPs.

NPs	*K* (×10^3^ M^−1^)	Δ*H* (×10^4^ kcal·mol^−1^)	Δ*S* (cal·mol^−1^.K^−1^)	*N*
L-PAV-AuNPs	4.04	−5.655	−173	32.1
D-PAV-AuNPs	9.86	−1.484	−31.5	115
