# Supplementary material for: Surface-anchored poly(acryloyl-L(D)-valine) with enhanced chirality-selective effect on cellular uptake of gold nanoparticles
Source: Sci Rep. 2016 Aug 17;6:31595. doi: 10.1038/srep31595 (PMC4987644; doi:10.1038/srep31595)
Supplement: Supplementary Information [file srep31595-s1.doc]

**Supporting Information for**

**Surface-anchored poly(acryloyl-L(D)-valine) with enhanced chirality-selective effect on cellular uptake of gold nanoparticles**

Jun Deng, Sai Wu, Mengyun Yao, Changyou Gao*

MOE Key Laboratory of Macromolecular Synthesis and Functionalization, Department of Polymer Science and Engineering, Zhejiang University, Hangzhou 310027, China.

*Corresponding author.

Email: [cygao@mail.hz.zj.cn](mailto:cygao@mail.hz.zj.cn)

Fax: +86-571-87951108

**Materials and methods**

**Materials**

L-valine, D-valine, 1-butanethiol, carbon disulfide (CS_2_), ethyl 2-bromopropionate, thioacetic acid and acryloyl chloride were purchased from Aladdin company. Triethylamine (TEA), dimethyl formamide (DMF) and dichloromethane (DCM) were obtained from Sinopharm Chemical Regent Co., Ltd, and were vacuum-distilled prior to use. If there was no special mention, other chemical regents were purchased from Sinopharm Chemical Regent Co., Ltd and without purification.

**Synthesis and characterization of** **methyl 2-(butylthiocarbonothioylthio)propanoate (MCEBTTC)**

Synthesis of RAFT agent of MCEBTTC.

The MCEBTTC was synthesized according to the literature procedures [^1^](#_ENREF_1). Briefly, CS_2_ (6.18 mL, 0.103 mol) in DCM (50 mL) was added to a stirred solution of 1-butanethiol (10 mL, 0.093 mol) and TEA (14.3 mL, 0.103 mol) in DCM (100 mL) under nitrogen via a constant pressure drop funnel over a period of 30 min at 0 ^o^C. After full addition, the solution was allowed to stir at room temperature for 1 h. Then methyl-2-bromopropionate (MBP, 1.46 mL, 0.103 mol) in DCM (50 mL) was added through a constant pressure funnel over a period of 30 min and allowed to stir for 2 h. The solution was dried and re-dissolved in Et_2_O and filtered to remove the TEA-HBr salt. The solution was then washed with cold 10 % HCl solution (3 × 50 mL) and Milli-Q water (3 × 50 mL), and dried over anhydrous MgSO_4_. A yellow, oily substance remained after ether removal. The substance was purified by column chromatography (20:1 pentane/ethyl acetate, second band) and characterized by ^1^H-NMR. ^1^H-NMR (CDCl_3_) CH_3_ δ 0.92 (tri, 3H), CH_2_ δ 1.43 (m, 2H), CH_3_ δ 1.62 (d, 3H), CH_2_ δ 1.65 (q, 2H), CH_2_ δ3.36 (tri, 2H), CH_3_ δ 3.73 (s, 3H), CH δ 4.84 (q, 1H). Moreover, the oily substance was characterized by a Bruker Esquire 3000 plus ion trap mass spectrometer (Brucker-Franzen Analytik GmbH, Bremen, Germany): [M+H]^+^: 253, [M+Na]^+^: 275 and [M+K]^+^: 291.1 for MCEBTTC.

**Synthesis and characterization of** **acryloyl-L(D)-valine monomers**

Synthesis of acryloyl-L(D) -valine monomers.* represents chiral center.

The acryloyl-L(D)-valine monomers were synthesized according to literature procedures with modification [^2^](#_ENREF_2). 5.86 g (0.05 mol) L(D)-valine and 4 g (0.1 mol) NaOH were added into a 250 mL one-neck round bottom flask equipped with a magnetic stirrer, and were dissolved in 20 mL water. The solution was then cooled to ~5 ^o^C. 4.52 g (0.05 mol) acryloyl chloride was added slowly to the solution below 8 ^o^C. After complete addition the solution was allowed to react for another 2 h. The mixtures were then neutralized with 8.5 mL of concentrated HCl, resulting in the formation of white precipitates. The precipitates were extracted with ethyl acetate (4 × 30 mL), and the organic phase was dried with anhydrous sodium sulphate. After filtering and evaporation to remove most part of the solvent, the concentrated solution was stored overnight for crystallization. Colorless granular crystal was obtained. The crystal monomers were characterized by ^1^H-NMR (CD_3_OD, 600MHz DD2, Agilent). CH_3_ δ 0.89 (d, 6H), CH δ 2.11 (mult,1H), CH δ 4.31 (d, 1H), CH δ 5.58 (tri, 1H), CH_2_ δ 6.17, 6.28 (m, 2H). The crystal monomers were further characterized by a Bruker Esquire 3000 plus ion trap mass spectrometer: [M+H]^+^: 172, [M+Na]^+^:193.7 and [M+K]^+^: 209.7 for acryloyl-L-valine; [M+H]^+^ 172.4, [M+Na]^+^:193.9 and [M+K]^+^: 209.7 for acryloyl-D-valine.

**Size distribution**

Figure S1. A size distribution calculated for (a) L-MAV-AuNPs, (b) D-MAV-AuNPs, (c) L-PAV-AuNPs and (d) D-PAV-AuNPs from TEM images (based on random counting of more than 500 particles). These diameters were used to calculate the total particle concentration using ICP-MS measurements.

**Thermogravimetric analysis (TGA)**

The density of L(D)-MAV and L(D)-PAV grafted on AuNPs was measured by TGA (Q50 V20.13 Build 39) (Figure S2)**.** The analysis was performed from 50 °C up to 800 °C at a fixing heating rate of 10 °C·min^−1^ under a continuous flux of nitrogen.

Figure S2. TGA curves of (a) L-MAV-AuNPs and D-MAV-AuNPs; (b) L-PAV-AuNPs and D-PAV-AuNPs.

**Conjugate characterization**

Figure S3. Characterization of MAV-AuNPs and PAV-AuNPs stability in cell culture medium. SPR spectra of (a) citrate-AuNPs, L(D)-MAV-AuNPs and L(D)-PAV-AuNPs in water, (b) L-MAV-AuNPs and D-MAV-AuNPs in water and 10 % FBS/DMEM, and (c) L-PAV-AuNPs and D-PAV-AuNPs in water, DMEM and 10% FBS/DMEM. Here phenol red-free DMEM was used.

The subsequent investigation of the conjugates revealed the extent of the ligand and FBS concentration specific differences. Conjugate stability was monitored through absorption measurement of the particle SPR, a parameter determined by coherent oscillation of conduction electrons in the AuNP surface that are exquisitely sensitive to their local dielectric environment [^3^](#_ENREF_3). The SPR of the gold NPs was determined by recording the absorbance of gold NPs (400-700 nm) in water and DMEM containing 10 % FBS or not on a UV-vis spectrophotometer (Shimadzu UV2550). Each spectrum was an average of those of 3 individual samples recorded twice. Compared to citrate-AuNPs, SPR peak of the L(D)-MAV-AuNPs and L(D)-PAV-AuNPs was red-shifted for 13 nm and 4 nm, respectively (Figure S3a). When being incubated in 10 % FBS/DMEM, the SPR peak of the PAV-AuNPs was kept at 521 nm without change, whereas the SPR peak of the MAV-AuNPs was slightly blue-shifted (9 nm) (Figure S3b). The SPR peak of the PAV-AuNPs from DMEM was red-shifted slightly with a value of 4 nm. No significant peak broadening was observed, suggesting good dispersion (Figure S3c). These results show that the PAV-AuNPs were very stable in all the above mediums. It has to be pointed out that the DMEM used here did not contain the phenol red.

**TEM**

Figure S4. Representative TEM images of (a, e) L-MAV-AuNPs, (b, f) D-MAV-AuNPs, (c, g) L-PAV-AuNPs, and (d, h) D-PAV-AuNPs. The NPs were incubated in (a-d) water and (e-h) 10 % FBS/DMEM, respectively. Scale bar is 100 nm.

The diameters of MAV-AuNPs and PAV-AuNPs before and after being incubated in 10 % FBS/DMEM were further characterized by using the transmission electron microscopy (TEM, H-7650). Briefly, the NPs (MAV-AuNPs and PAV-AuNPs) were incubated in water and 10 % FBS/DMEM at 37 ^o^C for 12 h, respectively. Then, the NPs solutions were diluted with water, and 5 *μ*L of diluted NPs solution was applied on a carbon-coated copper grid and dried under atmosphere overnight. For NPs incubated in 10 % FBS/DMEM, 5 *μ*L water was applied on a carbon-coated copper grid for 1 min, and then a filter paper was used to carefully adsorb the water. This process was repeated twice to remove the ions. Phenol-red free DMEM was used in this study.

**Cytoviability**

To determine the viability of HepG2 or A549 cells, the cells were plated at a density of 5×10^4^ cells/cm^2^ in a 24-well plate and cultured for 24 h. The medium was replaced with fresh one containing the L(D)-MAV-AuNPs or the L(D)-PAV-AuNPs (0.9 mL for each well) with a given Au concentration (40 *μ*g/mL and 80 *µ*g/mL). After treatment for another 24 h, 100 *μ*L 5 mg/mL MTT solution was added to each well, and the cells were further cultured at 37 °C for 3 h. The dark blue formazan crystals generated by the mitochondria dehydrogenase in live cells were dissolved with dimethyl sulfoxide. After the sample was centrifuged at 12000 g/min for 5 min, the absorbance of supernatant was measured by a microplate reader (MODEL 680, Bio Rad) at 570 nm. The results were shown in Figure S5.

Figure S5. Influence of PAV-AuNPs concentration on (a) A549 and (b) HepG2 cell viability. The cells were cultured with 40 *µ*g·mL^-1^ or 80 *µ*g·mL^-1^ PAV-AuNPs for 24 h. Influence of pharmacological inhibitors on (c) A549 and (d) HepG2 cell viability. The cells were cultured with pretreatment by amantadine-HCl (Aman, 1 mM, inhibitor of clathrin-mediated endocytosis), genistein (Ge, 100 *µ*M, inhibitor of caveolae-mediated endocytosis), amiloride-HCl (Amilo, 2 mM, inhibitor of macropinocytosis), NaN_3_ (0.1 % (w/v), inhibit energy-dependent process) or cytochalasin D (CytD, 10 *μ*g·mL^−1^, inhibitor of cytoskeleton) for 1 h. Then the medium was replaced with fresh one containing the L(D)-PAV-AuNPs with an Au concentration of 50 *µ*g/mL. The cells without pharmacological inhibitors treatment were used as control. * and ** indicate significant difference at *p* < 0.05 level and *p<*0.01 vs respective particle-free control, respectively.

**Cellular uptake**

Figure S6. Internalized amount of (a) the L(D)-MAV-AuNPs and L(D)-PAV-AuNPs by HepG2 cells in 10 % FBS/DMEM and (b) the PAV-AuNPs pretreated with 1 mg/mL D-valine or D-PAV (*M*_w_: 18743 Da) in 10 % FBS/DMEM, and (c) the PAV-AuNPs incubated in serum-free DMEM at an Au concentration of 50 *µ*g/mL for 24 h. (d) Influence of pharmacological inhibitors on uptake of L-PAV-AuNPs and D-PAV-AuNPs. The cells were cultured without or with pretreatment by amantadine-HCl (Aman, 1 mM, inhibitor of clathrin-mediated endocytosis), genistein (Ge, 100 *µ*M, inhibitor of caveolaemediated endocytosis), amiloride-HCl (Amilo, 2 mM, inhibitor of macropinocytosis), cytochalasin D (CytD, 10 *μ*g· mL^−1^, inhibitor of cytoskeleton), or NaN_3_ (inhibit energy-dependent process) for 1 h, and then cultured with PAV-AuNPs for another 4 h. * and ** indicate significant difference at *p* < 0.05and *p*<0.01, respectively.

The amount of L(D)-MAV-AuNPs or L(D)-PAV-AuNPs internalized by HepG2 cells was elevated by ICP-MS. The result was presented in Figure S6a. Influence of D-valine, serum adsorption, and pharmacological inhibitors on uptake of L(D)-PAV-AuNPs was shown in Figure S6 b, c, d, respectively.

Figure S7. Concentration-dependent cellular uptake of PAV-AuNPs NPs. The HepG2 cells were incubated with 10, 50 and 100 µg·mL^-1^ PAV-AuNPs for 24 h. ** represent significant difference at *p* < *p*<0.01 level.

**Protein adsorption**

The amount of proteins adsorbed on NPs was measured by the Micro Bicinchoninic Acid (µBCA ) assay (Pierce, USA) according to the manual instructions. Briefly, the PAV-AuNPs were incubated in 10 % FBS/DMEM and 4 mg/mL bovine serum albumin (BSA)/DMEM. The samples were shaken at 37 ^o^C for 24 h. After the samples were centrifuged at 10 ^o^C for 40 min, the supernatants were discarded. The PAV-AuNPs were re-dispersed in PBS by gentle shaking, and centrifuged again. Totally 5 washes were performed to remove the free serum proteins and BSA. The protein-adsorbed PAV-AuNPs were then treated with 5% sodium dodecylsulphate (SDS) to release the proteins, whose amount was quantified by using the *μ*BCA assay regent kits. The results were shown in Figure S8.

Figure S8. The density of adsorbed proteins on L-PAV-AuNPs and D-PAV-AuNPs from 4 mg·mL^-1^ BSA and 10% FBS/DMEM measured by µBCA, respectively.

**NP distribution**

The distribution of L(D)-PAV-AuNPs in HepG2 cells obtained by TEM is shown in Figure S9.

Figure S9. Representative TEM micrographs of sectioned HepG2 cells after being cultured with (a, a1 and a2) L-PAV-AuNPs and (b, b1 and b2) D-PAV-AuNPs at the same Au concentration of 50 *µ*g/mL in 10 % FBS/DMEM, showing subcellular localization of the internalized NPs, respectively. The arrow heads indicate the PAV-AuNPs, and the black arrows indicate the amplification regions. Ly: Lysosome; N: Nucleus.

**References**

1． Truong, N. P., Jia, Z., Burges, M., McMillan, N. A. & Monteiro, M. J. Self-catalyzed degradation of linear cationic poly (2-dimethylaminoethyl acrylate) in water. *Biomacromolecules* **12**, 1876-1882 (2011).

2． Ezell, R. G., Gorman, I., Lokitz, B., Ayres, N. & McCormick, C. L. Stimuli‐responsive ampholytic terpolymers of N‐acryloyl‐valine, acrylamide, and (3‐acrylamidopropyl) trimethylammonium chloride: Synthesis, characterization, and solution properties. *J Polym Sci Polym Chem* **44**, 3125-3139 (2006).

3． Eustis, S. & El-Sayed, M. A. Why gold nanoparticles are more precious than pretty gold: Noble metal surface plasmon resonance and its enhancement of the radiative and nonradiative properties of nanocrystals of different shapes. *Chem Soc Rev* **35**, 209-217 (2006).
